# Changes in hemoglobin and clinical outcomes drive improvements in fatigue, quality of life, and physical function in patients with paroxysmal nocturnal hemoglobinuria: post hoc analyses from the phase III PEGASUS study

**DOI:** 10.1007/s00277-022-04887-8

**Published:** 2022-07-23

**Authors:** David Cella, Sujata P. Sarda, Ray Hsieh, Jesse Fishman, Zalmai Hakimi, Kate Hoffman, Mohammed Al-Adhami, Jameel Nazir, Katelyn Cutts, William R. Lenderking

**Affiliations:** 1 Northwestern University, Chicago, IL USA; 2 Apellis Pharmaceuticals, Inc., Waltham, MA USA; 3 Evidera, 7101 Wisconsin Avenue, Suite 1400, Bethesda, MD 20814 USA; 4 Sobi, Stockholm, Sweden

**Keywords:** Bilirubin, EORTC QLQ-C30, FACIT-F, Hemoglobin, Patient-reported outcomes

## Abstract

Paroxysmal nocturnal hemoglobinuria (PNH) is a rare, chronic, acquired, hematologic, life-threatening disease characterized by thrombosis, impaired bone marrow function, and complement-mediated hemolysis. The PEGASUS phase III clinical trial demonstrated superiority of pegcetacoplan over eculizumab regarding improvements in hemoglobin levels in patients with suboptimal response to prior eculizumab treatment. The objective of this post hoc analysis was to compare the patient-reported outcome (PRO) response rates observed among PEGASUS participants and the relationships between their PRO scores with clinical and hematological parameters. Data from the 16-week randomized, controlled (1:1 to pegcetacoplan or eculizumab) period of the PEGASUS trial included comparisons of weekly PRO measurements taken using the Functional Assessment of Chronic Illness Therapy-Fatigue (FACIT-F) scale and the European Organization for Research and Treatment of Cancer Quality of Life Questionnaire Core-30 (EORTC QLQ-C30) scales. A clinically meaningful FACIT-F response was defined as an increase from baseline of ≥5 points. Convergent validity was assessed using conventional threshold correlations between FACIT-F, EORTC QLQ-C30, and laboratory parameters. A clinically meaningful improvement in FACIT-F score was seen in 72.2% of pegcetacoplan-treated patients compared to 22.9% of eculizumab-treated patients. At week 16, the FACIT-F total score correlated with hemoglobin levels (r=0.47, *p*< 0.0001), absolute reticulocyte count (r=−0.37, *p*<0.01), and indirect bilirubin levels (r=−0.25, *p*<0.05). Clinically meaningful improvements in pegcetacoplan-treated patients were also observed for multiple EORTC scales. Fatigue and other self-reported outcomes were correlated with clinically meaningful improvements in clinical and hematological parameters. Clinical trial registration: NCT03500549

**Supplementary Information:**

The online version contains supplementary material available at 10.1007/s00277-022-04887-8.

## Introduction

Paroxysmal nocturnal hemoglobinuria (PNH) is a rare, chronic, acquired, hematologic, life-threatening disease characterized by thrombosis, impaired bone marrow function, complement-mediated hemolysis, and anemia [1]. The clinical manifestations of PNH are associated with significant impairments in physical and social functioning as well as global health status [2]. Frequently reported symptoms include fatigue, dyspnea, hemoglobinuria, and pain [2, 3]. These symptoms, among a variety of others reported, may significantly reduce the health-related quality of life (HRQoL) and productivity of patients with PNH [3]. Instruments such as the Functional Assessment of Chronic Illness Therapy-Fatigue (FACIT-F) scale and European Organisation for Research and Treatment of Cancer Quality of Life Questionnare-C30 (EORTC QLQ-C30) have been developed to assess the effects of treatment on outcomes such as fatigue and physical function from the patient’s perspective. Assessments of patient-reported outcomes (PRO) are particularly important in disease states such as PNH, where patients’ HRQOL is largely affected by symptoms of disease progression.

Prior to 2007, the main treatment options for PNH were supportive and included blood transfusions, erythropoiesis-stimulating agents, corticosteroids, anabolic steroids, iron therapy, and thrombosis prophylaxis [4, 5]. The development of terminal complement C5 inhibitors, such as eculizumab and ravulizumab, has provided highly effective therapies that control intravascular hemolysis in patients with PNH. However, these therapies appear to have some limitations. For example, between 27 and 39% of eculizumab- or ravulizumab-treated patients may experience breakthrough hemolysis due to insufficient complement inhibition [6–9]. In addition, patients remain anemic and transfusion-dependent despite treatment with eculizumab and ravulizumab; one study reported that approximately 50% of patients treated with eculizumab and up to 40% of patients treated with ravulizumab had at least one transfusion in the previous year [10–13]. Additionally, eculizumab- and ravulizumab-treated patients have also reported considerable loss of work-related productivity, greatly diminished ability to work, and limitations in their usual activities [9, 14].

Pegcetacoplan is a pegylated molecule that targets the complement C3 protein, thereby controlling both intravascular and extravascular hemolysis [15] and regulating the subsequent activation of effector functions in the complement cascade. Primary results from the phase III PEGASUS trial (NCT03500549) that assessed the efficacy and safety of pegcetacoplan compared to eculizumab demonstrated that pegcetacoplan was superior to eculizumab in change from baseline to week 16 in hemoglobin level, the primary endpoint of the study, in PNH patients with hemoglobin levels <10.5 g per deciliter despite eculizumab therapy. Moreover, patients treated with pegcetacoplan experienced a substantial reduction in fatigue at week 16 compared to baseline, a secondary endpoint, that was measured using the FACIT-F scale [16]; this reduction was maintained at 48 weeks [17]. Evaluation of another secondary endpoint, EORTC QLQ-C30, also showed improvements in the pegcetacoplan group across all scales, with the exception of diarrhea.

Although the initial analysis of the PEGASUS study reported the primary and secondary endpoints, including FACIT-F and the EORTC QLQ-C30, additional information about the study participants and their reported outcomes were available. FACIT-F and EORTC QLQ-C30 were measured weekly along with weekly laboratory measurements of hemoglobin, absolute reticulocyte count, and indirect bilirubin. Collectively, these have not been previously reported at each time point. Further, our analysis sought to evaluate different thresholds of clinical response, or clinically important differences. Finally, there is little known about the associations between laboratory measures that are commonly evaluated by clinicians of patients with PNH and their associations with patient-reported fatigue. By evaluating the associations between laboratory and patient-reported parameters, clinicians and patients can better understand and track the impact of PNH on daily life. In this post hoc analysis of the Phase III PEGASUS trial data, we compared PRO response rates observed among PEGASUS participants and the relationships between their PRO scores and clinical and hematological parameters.

## Methods

### PEGASUS phase III randomized controlled trial study design

**Fig. 1:**
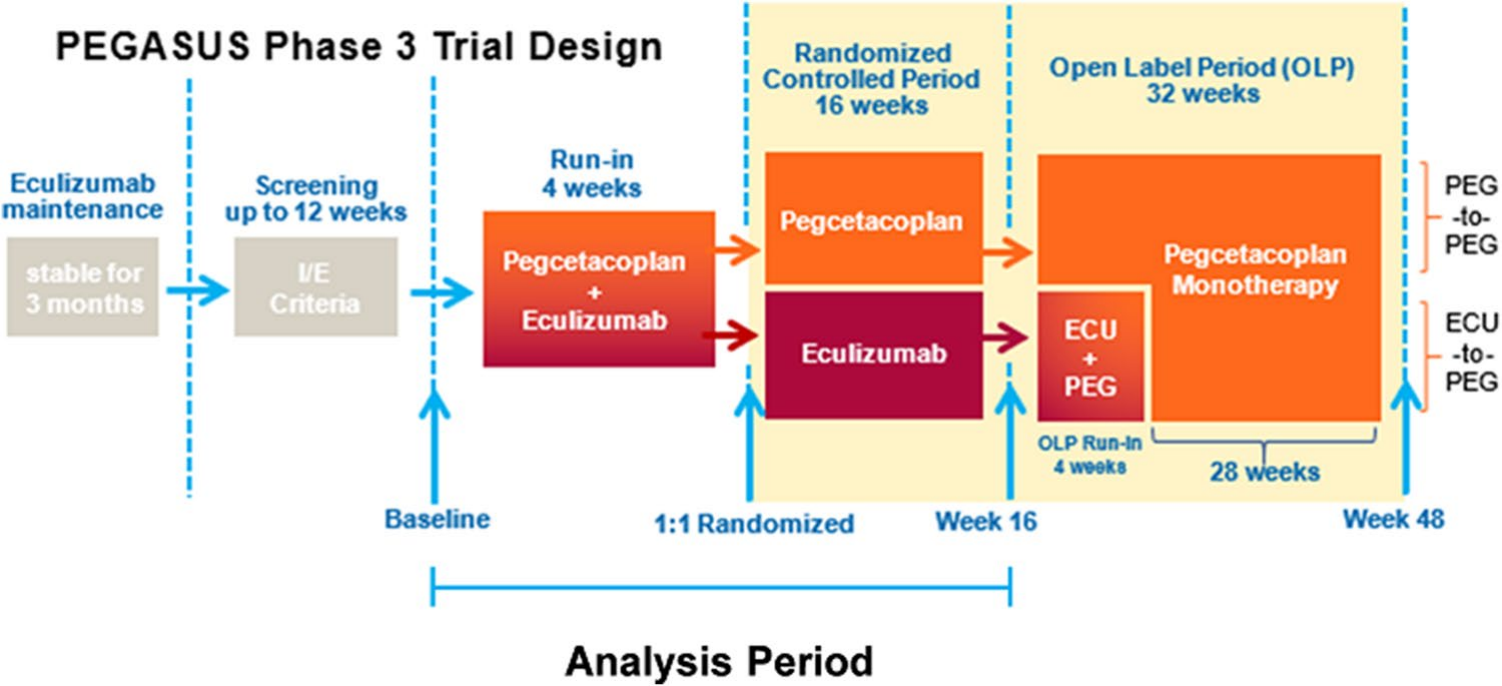
PEGASUS study design. ECU, eculizumab; I/E, inclusion/exclusion; PEG, pegcetacoplan; SC, subcutaneous

The PEGASUS study design and results have been previously published [16]. Briefly, 80 participants were randomized across 44 multinational sites. The PEGASUS protocol was approved by ethics committees at participating sites, and all patients provided written informed consent. Eligible patients in PEGASUS included men and women ≥18 years of age with a diagnosis of PNH by high-sensitivity flow cytometry who had hemoglobin levels <10.5 g/dL, while receiving stable doses of eculizumab for ≥3 months before screening. The trial treatment period consisted of three parts: (1) a 4-week run-in period in which all patients continued their current dose of eculizumab, with the addition of twice weekly subcutaneous pegcetacoplan 1080 mg; (2) a 16-week randomized, controlled period in which patients were randomized 1:1 to eculizumab or pegcetacoplan as monotherapies; and (3) a 32-week open-label period in which all patients received pegcetacoplan (with eculizumab for the first 4 of those 32 weeks (Fig. 1)). The primary outcome of the randomized controlled PEGASUS trial was the change in hemoglobin level from baseline to week 16 [16]. Secondary outcomes included FACIT-F total score (version 4) and EORTC QLQ-C30 (version 3) total scores at week 16.

In the present analysis, a sample size calculation was not performed, as these analyses examine PROs by different definitions of responsiveness and their correlations between laboratory parameters, which were not preplanned at the start of PEGASUS. The current analysis also reports on the 16-week head-to-head period, as the crossover study design did not permit a comparative analysis in the 32-week open label period in which all participants received only pegcetacoplan.

### Patient-reported outcome instruments

The FACIT-F is a 13-item tool that measures an individual’s level of fatigue during their usual daily activities over the past week (https://www.facit.org/measures/FACIT-F) [18]. The FACIT-F has been validated in a PNH population where a qualitative content validity study was completed in 29 patients from four countries (United States [US], United Kingdom [UK], Spain, and France) [19]. Each item is rated on a 5-point (0–4) rating scale. The total score range is therefore 0 to 52, with most items reverse-scored so that higher scores indicate less fatigue. The FACIT-F was measured weekly for 16 weeks following randomization.

The EORTC QLQ-C30 questionnaire is a 30-item patient-reported outcome that incorporates nine multi-item scales including five functional scales (physical, role, cognitive, emotional, and social), three symptom scales (fatigue, pain, and nausea and vomiting), and global health and quality-of-life scales [20]. The remaining single items assess additional symptoms commonly reported by cancer patients such as dyspnea, appetite loss, sleep disturbance, constipation, and diarrhea, as well as the perceived financial impact of disease burden and treatment. Items are rated on 4-point rating scales, except for two items on global health status/QoL, which use seven-point rating scales. Scores range from 0 to 100; a high score for a functional scale denotes a high level of functioning, whereas a high score for a symptom scale/single item represents a high level of symptomatology [20]. The EORTC QLQ-C30 was measured weekly for 16 weeks following randomization.

Paper-based versions of the FACIT-F and EORTC QLQ-C30 questionnaire were self-administered by patients at each clinic visit.

### Post hoc analysis

In this manuscript, we report post hoc comparisons of FACIT-F and EORTC QLQ-C30 patient-reported response rates from the 16-week randomized, controlled period of the PEGASUS trial as well as the relationships between PRO scores and clinical and hematological parameters.

Responsiveness, the ability of our analysis to detect underlying change, was included to evaluate the extent to which EORTC QLQ-C30 and FACIT-F detected a true change among the patients known to have a change in their clinical status [21]. For responsiveness measures [21] of the FACIT-F and EORTC QLQ-C30, participants were grouped into the following anchors from baseline to week 16 by (1) degree of hemoglobin levels improvement: <1 g/dL (“non-responders”), ≥1 to <2 g/dL (“partial responders”), and ≥2 g/dL (“responders”), (2) a decrease in absolute reticulocyte count (≥median [70x10^9^ cells/L] vs. <median [70x10^9^ cells/L]), and (3) a decrease in indirect bilirubin levels (≥median 7.6 μmol/L vs. <median 7.6 μmol/L).

### Statistical analyses

Data are presented for the full analysis set (all patients randomized to treatment who received ≥1 dose of study drug and who had ≥1 post-baseline assessment). Importantly, in the previous publication of the PEGASUS study results [16], transfusions were classified as intercurrent events that could confound the primary outcome of change in hemoglobin and, consequently, data after the first transfusion were censored among those receiving a transfusion. Numerous factors are associated with hemoglobin variability including red blood cell transfusions, infections, and inflammation, which provides the rationale for the analysis recommendations from the FDA and which resulted in excluding transfused patients from the primary outcome analysis [22]. In contrast to previous results from the PEGASUS study, the analyses presented here include all available patients regardless of intercurrent events. Thus, the entire available patient sample was used when evaluating the association between the primary efficacy endpoint of hemoglobin and FACIT-F or EORTC QLQ-C30.

In this paper, we use the term clinically important difference (CID), which is best estimated as a range of the PRO score to reflect a change that is meaningful to patients. In this case, we set the CID for FACIT-F as ≥5 points; a value that comfortably exceeds the likely minimal clinically important difference.

Descriptive statistics were used to characterize the patient population. Between-treatment group comparisons were performed using a mixed effect model for repeated measures (MMRM). The model included fixed categorical effects for treatment group, study visit, stratification variables, and the study visit-by-treatment group interaction, as well as the continuous, fixed covariate of baseline parameters level.

For convergent validity comparisons, correlations between FACIT-F scores and the EORTC QLQ-C30 domain and total scores with hemoglobin levels, absolute reticulocyte count, and bilirubin levels were examined using Spearman correlations. The strength of the correlation was interpreted using the following guidance, where the absolute value of correlation coefficient values of 0.2–0.3 were generally regarded as “weak,” 0.3–0.5 as “moderate,” and >0.5 as “strong” [23].

All statistical tests were two-sided and used a significance level of 0.05, unless otherwise noted. All analyses were performed using SAS version 9.4 (Cary, North Carolina, US).

## Results

Patient demographics and clinical characteristics were generally balanced between the pegcetacoplan and eculizumab treatment groups (Table 1). Patients in the pegcetacoplan treatment group had a mean (standard deviation [SD]) age of 50.2 (16.3) years and 34.1% were male. Patients in the eculizumab treatment group had a mean age of 47.3 (15.8) years; 43.6% were male and 64.1% were white. For the FACIT-F, the overall compliance rate was 91% at week 16. Five pegcetacoplan-treated patients and two eculizumab-treated patients did not complete the PRO at week 16.

**Table 1:** Patient demographic and clinical characteristics

Characteristic	Total (*N*=80)	
	PEG (*N*=41)	ECU (*N*=39)
Age in years (mean, SD)	50.2 (16.3)	47.3 (15.8)
Sex (n, % male)	14 (34.1)	17 (43.6)
BMI (mean, SD)	26.7 (4.3)	25.9 (4.3)
Race (n, % yes)		
White	24 (58.5)	25 (64.1)
Asian	5 (12.2)	7 (17.9)
Black or African American	2 (4.9)	0 (0.0)
Other	0 (0.0)	1 (2.6)
Missing	10 (24.4)	6 (15.4)
Ethnicity (n, % yes Hispanic or Latino)	2 (4.9)	1 (2.6)
Height in centimeters (mean, SD)	167.7 (10.3)	169.1 (8.7)
Weight in kilograms (mean, SD)	75.9 (18.8)	74.6 (16.6)

*BMI*, body mass index; *ECU*, eculizumab; *PEG*, pegcetacoplan; *SD*, standard deviation

The mean (SD) FACIT-F total score at baseline was 32.2 (11.4) for pegcetacoplan-treated patients and 31.6 (12.5) for eculizumab-treated patients. The change in mean FACIT-F total score from baseline to week 16 was reported previously [16]. At week 16, least squares change from baseline reported as mean (standard error [SE]) in FACIT-F total score was significantly higher for patients in the pegcetacoplan treatment group (9.65 [1.64]) compared to those in the eculizumab treatment group (−1.7 [1.5]; *p*=<0.0001).

**Fig. 2:**
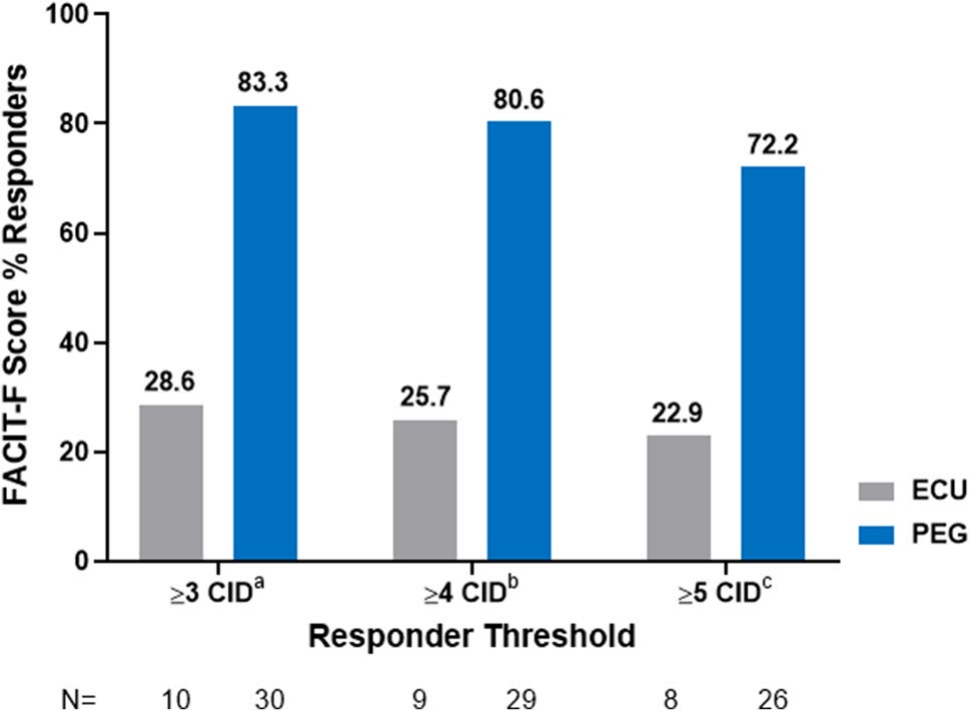
FACIT-F score % responders from baseline to week 16. CID, clinically important difference; ECU, eculizumab; FACIT-F, Functional Assessment of Chronic Illness Therapy–Fatigue; ICE, intercurrent events; PEG, pegcetacoplan. ^a^Mean Hg (g/dl) for ECU (≥3 CID) = −0.20 and PEG (≥3 CID) = 3.10; ^b^Mean Hg (g/dl) for ECU (≥4 CID) = −0.22 and PEG (≥4 CID) = 3.13; ^c^Mean Hg (g/dl) for ECU (≥5 CID) = −0.07 and PEG (≥5 CID) = 3.19. An increase of 3–5 points on the FACIT-F is in the range of published estimates of clinically important differences [24–28]

The proportion of FACIT-F Score responders by responder threshold from baseline to week 16 is shown in Fig. 2. A clinically meaningful individual improvement in FACIT-F score (≥5) was achieved in 72.2% of pegcetacoplan-treated patients compared to 22.9% of eculizumab-treated patients. Mean FACIT-F total scores at baseline and week 16 for each treatment group are shown in Supplemental Fig. 1A.

For the EORTC QLQ-C30, the overall compliance rate was 90% at week 16. Five pegcetacoplan-treated patients and three eculizumab-treated patients did not complete the PRO at week 16. A summary of baseline and change from baseline in EORTC QLQ-C30 functional domains and symptom scales at week 16 is shown in Table 2. Clinically meaningful improvements in pegcetacoplan-treated patients were observed for the following domains/scales: global health status/quality of life, physical functioning, role functioning, social functioning, fatigue, and dyspnea. Mean EORTC QLQ-C30 functional domains and symptom scales at baseline and week 16 for each treatment group are shown in Supplemental Fig. 1B-1E.

**Table 2:** Change from baseline in EORTC QLQ-C30 functional domains and symptom scales at week 16

	PEG (*N*=41)		ECU (*N*=39)	
	Baseline^a^	CFB at Wk 16^b^	Baseline^a^	CFB at Wk 16^b^
**Global Health Status/QoL**	56.30 (20.39)	**15.44** (3.05)	56.53 (20.24)	−3.83 (3.13)
**Functional scales**				
Physical functioning	71.38 (20.23)	**16.20** (2.34)	72.11 (20.14)	0.53 (2.44)
Role functioning	63.82 (29.56)	**16.15** (4.11)	59.65 (33.92)	−6.93 (4.25)
Emotional functioning	72.36 (25.38)	6.26 (3.39)	69.59 (22.67)	−2.65 (3.49)
Cognitive functioning	76.02 (24.45)	5.37 (3.21)	75.23 (25.95)	−8.87 (3.34)
Social functioning	69.51 (28.84)	**13.18** (3.40)	64.86 (32.82)	−0.16 (3.54)
**Symptom scales**				
Fatigue	49.59 (29.09)	−**22.34** (3.31)	50.29 (24.74)	−0.47 (3.41)
Nausea and vomiting	3.66 (8.75)	−0.10 (2.40)	5.26 (11.69)	6.13 (2.39)
Pain	19.51 (26.85)	1.31 (4.11)	15.79 (25.10)	9.48 (4.19)
Dyspnea	33.33 (27.90)	−**21.26** (3.61)	43.86 (32.05)	−3.86 (3.70)
Insomnia	32.52 (34.55)	−9.63 **(**3.61)	29.82 (29.80)	−5.53 (3.72)
Appetite loss	12.20 (17.88)	−4.68 (2.98)	13.16 (23.94)	2.06 (3.05)
Constipation	11.38 (20.56)	3.38 (2.81)	10.81 (22.30)	−5.60 (2.87)
Diarrhea	11.38 (23.11)	−0.33 (3.45)	11.71 (21.11)	8.27 (3.57)
Financial difficulties	18.70 (26.93)	−8.99 (3.62)	24.32 (37.39)	0.89 (3.84)

^a^Descriptive summary using all available data not censored for transfusion, mean (SD)

^b^MMRM model change from baseline to week 16, MMRM model includes all available data, LS mean CFB (SE)

The signs (+/-) for each scale reflect the amount of improvement. For example, functional scales with a positive value indicate improvement. In addition, bolded scores indicate a clinically meaningful change [16, 17] in EORTC QLQ-C30 scores, which is defined as a 10-point increase in global/functional scale and 10-point decrease in symptom scale/item

*CFB*, change from baseline; *ECU*, eculizumab; *EORTC QLQ-C30*, European Organization for Research and Treatment of Cancer Quality of Life Questionnaire – Core 30 Scale; *LS*, least squares; *PEG*, pegcetacoplan; *QoL*, quality of life; *Wk*, week

### Convergent validity

**Fig. 3:**
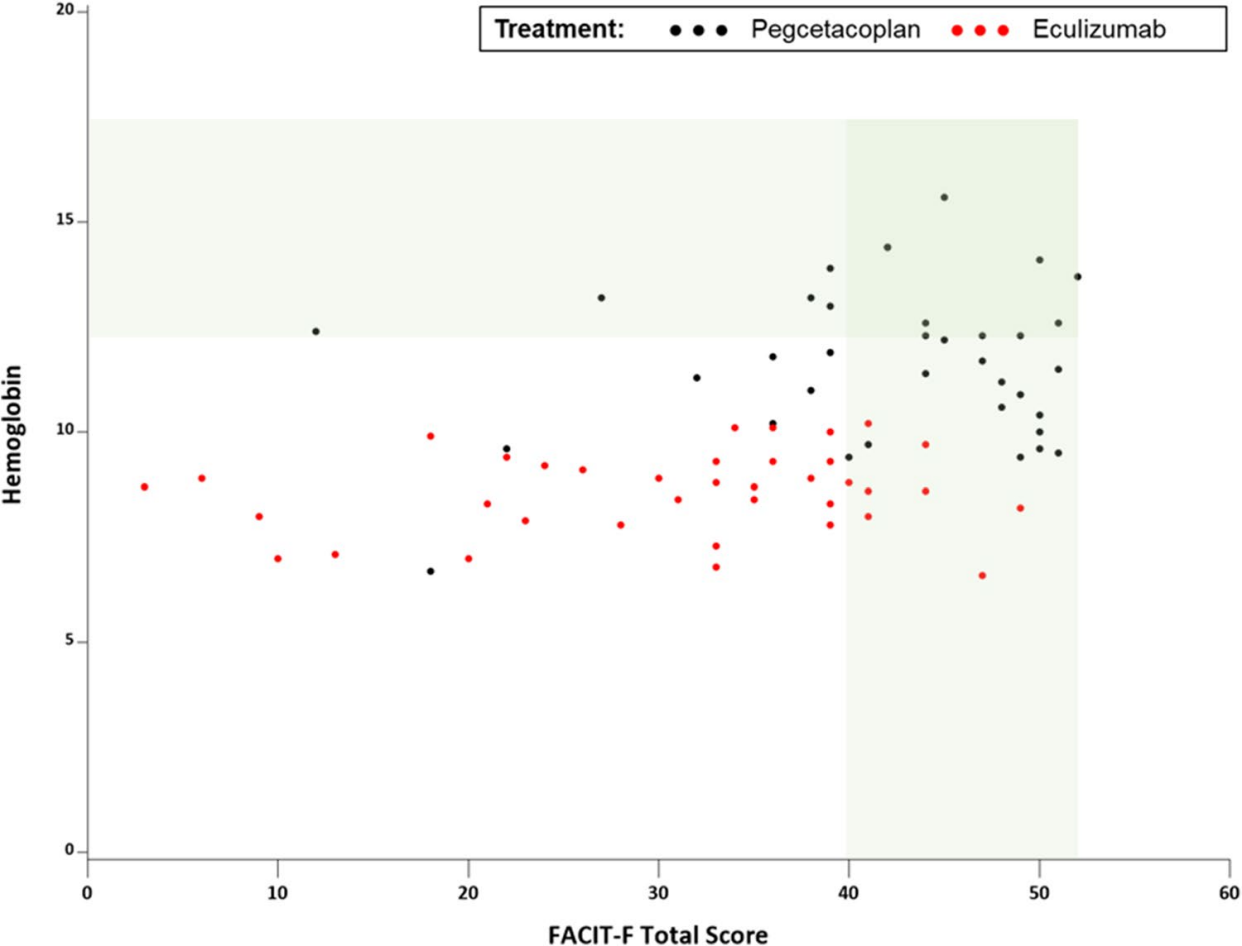
Hemoglobin and FACIT-F scores at week 16. FACIT-F, Functional Assessment of Chronic Illness Therapy–Fatigue. Green shaded bands represent normal score ranges for hemoglobin values and FACIT-F values

FACIT-F and EORTC QLQ-C30 correlations with hemoglobin levels, absolute reticulocyte count, and indirect bilirubin levels are displayed in Table 3. FACIT-F total scores correlated moderately with hemoglobin levels (r=0.47, *p*<0.0001; Fig. 3), and significantly, but less strongly, with absolute reticulocyte count (r=−0.37, *p*<0.01), and indirect bilirubin levels (r=−0.25, *p*<0.05).

**Table 3:** Correlation between FACIT-F and EORTC scores and clinical outcomes for both treatment groups

	Hemoglobin (r)	Reticulocyte count (r)	Indirect bilirubin (r)
FACIT-F			
Total score	0.47****	−0.37**	−0.25*
EORTC QLQ-C30			
Global Health Status/QoL	0.44****	−0.31**	−0.13
Function scale			
Physical function	0.45****	−0.28*	−0.26*
Symptom scale			
Fatigue	−0.39***	0.28*	0.18
Single item			
Dyspnea	−0.49****	0.38**	0.26*

Correlations - *p<0.05, **p<0.01, ***p<0.001, ****p<0.0001

*EORTC-QLQ-C30*, European Organization for the Research and Treatment of Cancer Quality of Life Questionnaire – Core 30 Scale; *FACIT-F*, Functional Assessment of Chronic Illness Therapy - Fatigue; *QoL*, quality of life; *r*, correlation coefficient

### Responsiveness

**Fig. 4:**
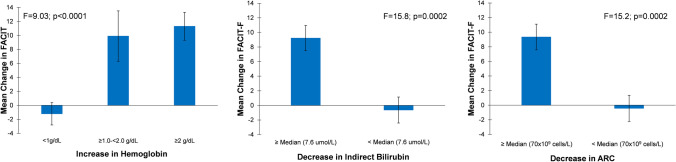
Patients with improvements in hemoglobin, indirect bilirubin, and ARC showed improvements in FACIT-F scores. ARC, absolute reticulocyte count; FACIT, Functional Assessment of Chronic Illness Therapy – Fatigue

When all patients, regardless of treatment, were grouped into various anchor measures, those with greater improvement in hemoglobin over 16 weeks occurred had the most improvement in fatigue (*p*<0.0001). The largest reduction in fatigue (11.3-point improvement in FACIT-F total score) was observed in the group with an increase in hemoglobin levels of ≥2g/dL (Fig. 4). Patients with a larger decrease in absolute reticulocyte count [≥ median (70x10^9^ cells/L)] and indirect bilirubin levels [≥ median (7.6 μmol/L)] had the largest reduction in fatigue (a 9.3-point improvement in FACIT-F total score [*p*=0.0002] for the absolute reticulocyte count group and a 9.22-point improvement for the indirect bilirubin levels [*p*=0.0002]) (Fig. 4).

**Fig. 5:**
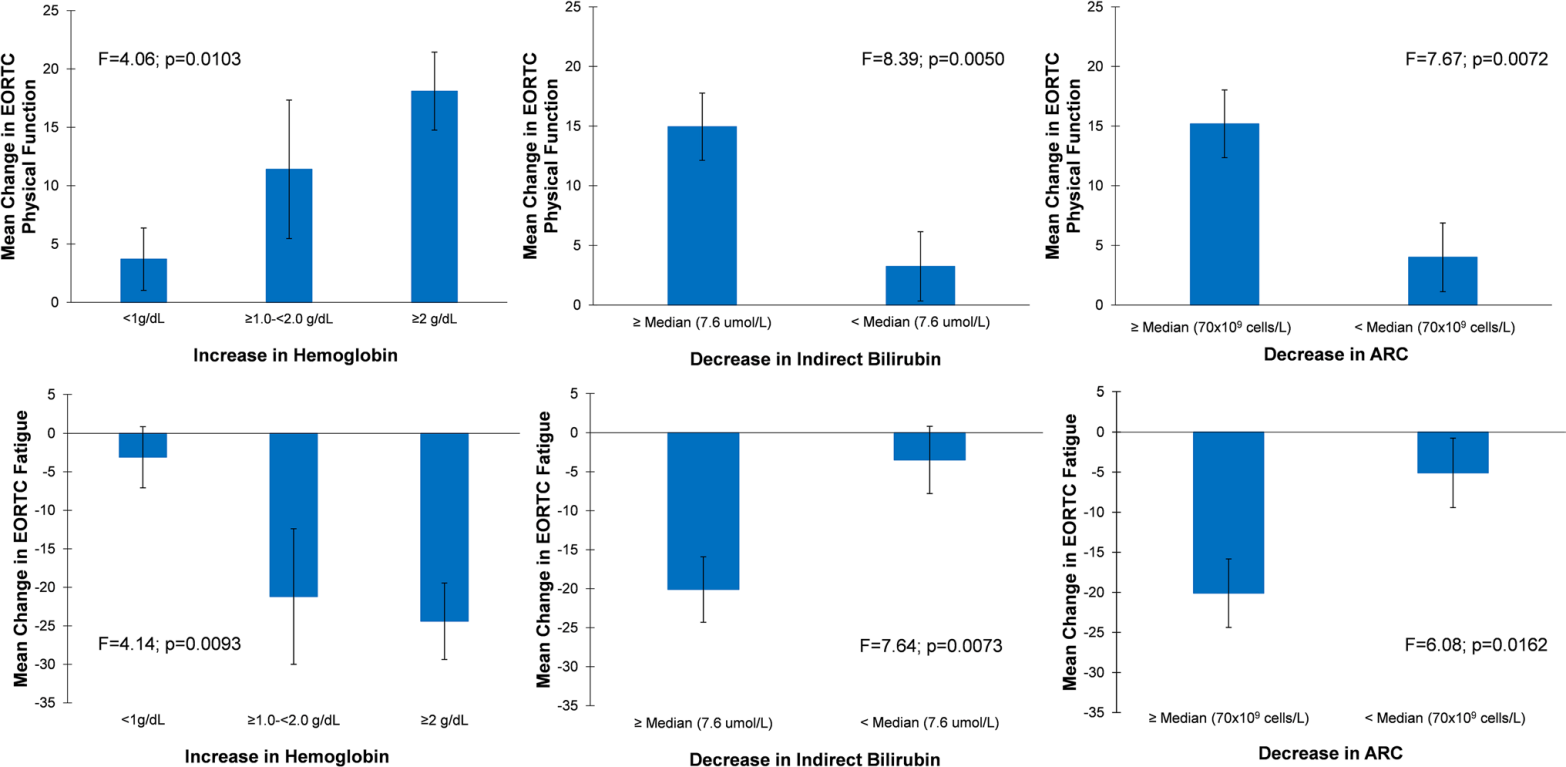
Patients with improvements in hemoglobin, indirect bilirubin, and ARC showed improvements in EORTC-QLQ-C30 physical functioning and fatigue scores. ARC, Absolute Reticulocyte Count; EORTC, European Organization for the Research and Treatment of Cancer

Similar results were observed for the EORTC QLQ-C30 across known groups including an increase in hemoglobin levels, a decrease in absolute reticulocyte counts, and a decrease in indirect bilirubin levels (Fig. 5).

## Discussion

This post hoc analysis used data from the PEGASUS study among all PNH patients randomized to receive treatment with pegcetacoplan or eculizumab. It was conducted to compare patient-reported fatigue and physical function response rates observed among PEGASUS participants and relationships between their PROs scores with clinical and hematological parameters.

When evaluating relationships between PROs and patient function, this analysis reports on correlated improvements of 5 points in FACIT-F score or 10 points in physical function (EORTC QLQ-C30), which were reported to be associated with hemoglobin level improvements among a large percentage of PNH patients treated with pegcetacoplan in this study. Importantly, across several endpoints, the magnitude of correlations was high, which may be of clinical importance as clinicians seek to evaluate fatigue and other PROs. This is integral to monitor patients’ response to treatment and their ability to perform activities of daily living.

Minimally important differences or change estimates in PNH or any other disease are context-dependent and subjective. Additionally, the minimally important clinical difference is no longer recommended by some experts and therefore CID was used for establishing a value for meaningful responses when evaluating the total PRO scores. To address some of the subjectivity, we used 5 points on the FACIT-F in this context to increase our confidence that patients classified as improved were indeed clinically improved. Smaller changes, such as 3 or 4 points, might also have been clinically important for some patients. The CID for FACIT-F varies and ranges between 3 and 5 are often reported in the literature as meaningful changes to patients [24–26, 29, 30]. In our study, based on weekly FACIT-F assessments, 83.3% of pegcetacoplan patients had a CID of ≥3 at week 16 which was nearly three times the response (28.6%) observed when compared to the eculizumab-treated.

The impact of nonfatal symptoms of PNH on a patient’s HRQoL is an integral area of focus when examining the effects of new treatments on disease progression. The EORTC QLQ-C30 and the FACIT-F are commonly used in evaluations of HRQoL among patients with cancer. Here, clinically meaningful improvements in pegcetacoplan-treated patients were observed for several EORTC QLQ-C30 functional domains and symptom scales including global health, physical functioning, dyspnea, and fatigue. FACIT-F and EORTC QLQ-C30 correlations with hemoglobin as well as reticulocyte count and indirect bilirubin, markers of extravascular hemolysis, were observed. The largest reduction in fatigue was observed in groups with greatest increases in hemoglobin, decreases in reticulocyte count, and decreases in indirect bilirubin. Of note, a median cut off was chosen for reticulocytes and bilirubin due to lack of established thresholds.

Previous findings have shown that despite treatment with eculizumab and ravulizumab for a period of up to 5 years, some patients remained severely anemic, were transfusion-dependent, and reported substantial fatigue [9, 16]. Here, we examined the relationship between patient-reported measures of fatigue and physical functioning with clinical and hematological parameters after treatment with pegcetacoplan or eculizumab. Fatigue is the most commonly reported symptom in patients with PNH and can have a negative impact on quality of life [2]. When examining the mean FACIT-F total score over the 16-week randomized period of the trial, pegcetacoplan treatment returned patients with PNH to a level comparable to that of the general population (approximately 43 in previous studies) [27, 28]. A correlation between pegcetacoplan-induced improvements in patient-reported fatigue, dyspnea, and improvements in hemoglobin levels was also observed, which is consistent with symptoms of fatigue and dyspnea accompanying anemia in patients with PNH [31]. These correlation-based study findings that demonstrate the association between FACIT-F scores and hemoglobin levels have also been observed in studies involving a variety of other patient populations, such as patients with nonmyeloid malignancy [32], chronic kidney disease [33], and primary hip arthroplasty [34], which may add some confidence that a correlation exists between the two measures.

Given the correlations between fatigue measured by FACIT-F and hemoglobin levels in this PNH population, it may be prudent for clinicians to consider which medical treatment can increase hemoglobin levels among PNH patients including those across a variety of hemoglobin ranges (e.g., even among >10 g/dL). According to these results, PNH patients may experience PRO improvements in fatigue and other symptoms from pegcetacoplan at various hemoglobin levels as treatment has been shown to lead to a reduction of transfusion requirements, and higher hemoglobin levels in the PEGASUS trial. Further, based on these correlation results, measurement of change in fatigue may predict changes in hemoglobin, changes that may warrant clinical exploration of the PNH patient.

Some limitations of this study should be considered when evaluating the results. The study included a relatively small sample size, although PNH is a rare hematologic disease which justifies this sample [35, 36]. In addition, the overall study design included an open-label period in which patients were aware of treatment allocation. The EORTC QLQ-C30 and the FACIT-F were originally developed for use in evaluations of HRQoL among people with cancer, with their validity extended to people with PNH. Other PRO questionnaires are available for patients with PNH that encompass additional aspects of the disease [37, 38]. Potential collection mode-related and non-response biases may have been introduced. Paper-based versions of the EORTC QLQ-C30 and the FACIT-F were administered to patients. Although there are several advantages to electronic versions of PROs (e.g., real-time data recording, immediate scoring, and reduction of human error), the implementation of traditional paper-based methods avoids the exclusion of certain patients who are less comfortable using electronic devices. Several studies have reported no significant differences between the two modes [39]. In addition, high compliance rates for completion of the EORTC QLQ-C30 and the FACIT-F were observed with very few dropouts across both treatment groups. Lastly, this study was not specifically designed for psychometric evaluation and results that are reported are based on a clinical trial population, so these may not be generalizable to other patient populations.

## Conclusions

Pegcetacoplan treatment resulted in a clinically meaningful reduction in fatigue levels by increasing hemoglobin levels at week 16 compared to eculizumab. Findings from this study also showed that fatigue and physical functioning outcomes were correlated with clinically meaningful improvements in clinical and hematological parameters. According to the convergent validity and responsiveness analyses, the FACIT-F and EORTC QLQ-C30 scales (global health status/quality of life, physical functioning, role functioning, social functioning, fatigue, and dyspnea) were also shown to be useful and valid patient-reported measures for assessing meaningful change in the treatment of PNH.

## Supplementary information

Supplemental Fig. 1. (A) Mean FACIT-F Total Score at baseline and week 16, (B) Mean EORTC-QLQ-C30 Global Health Status/QoL at baseline and week 16, (C) Mean EORTC-QLQ-C30 function scales at baseline and week 16, (D) Mean EORTC-QLQ-C30 symptom scales at baseline and week 16, and (E) Mean EORTC-QLQ-C30 individual items at baseline and week 16. Abbreviations: EORTC QLQ-C30, European Organization for Research and Treatment of Cancer Quality of Life Questionnaire – Core 30 Scale; ECU, eculizumab; FACIT-F, Functional Assessment of Chronic Illness Therapy–Fatigue; PEG, pegcetacoplan.

ESM 1 (PNG 12 kb)

High Resolution Image (TIF 24 kb)

ESM 2 (PNG 15 kb)

High Resolution Image (TIF 29 kb)

ESM 3 (PNG 25 kb)

High Resolution Image (TIF 59 kb)

ESM 4 (PNG 16 kb)

High Resolution Image (TIF 32 kb)

ESM 5 (PNG 26 kb)

High Resolution Image (TIF 55 kb)

## Data Availability

The data set used and analyzed during the current study is available from the corresponding author on reasonable request.

## References

[1] Hill A, DeZern AE, Kinoshita T, Brodsky RA (2017). Paroxysmal nocturnal haemoglobinuria. Nat Rev Dis Primers.

[2] Schrezenmeier H, Muus P, Socie G (2014). Baseline characteristics and disease burden in patients in the International Paroxysmal Nocturnal Hemoglobinuria Registry. Haematologica.

[3] Efficace F, Gaidano G, Breccia M (2015). Prevalence, severity and correlates of fatigue in newly diagnosed patients with myelodysplastic syndromes. Br J Haematol.

[4] Dmytrijuk A, Robie-Suh K, Cohen MH, Rieves D, Weiss K, Pazdur R (2008). FDA report: eculizumab (Soliris) for the treatment of patients with paroxysmal nocturnal hemoglobinuria. Oncologist.

[5] Luzzatto L (2016) Recent advances in the pathogenesis and treatment of paroxysmal nocturnal hemoglobinuria. F1000Res 23:5F1000 Faculty Rev-209. 10.12688/f1000research.7288.1. 10.12688/f1000research.7288.1 PMC4765720 26962442

[6] Hill A, Kelly RJ, Hillmen P (2013). Thrombosis in paroxysmal nocturnal hemoglobinuria. Blood.

[7] Nakayama H, Usuki K, Echizen H, Ogawa R, Orii T (2016). Eculizumab dosing intervals longer than 17 days may be associated with greater risk of breakthrough hemolysis in patients with paroxysmal nocturnal hemoglobinuria. Biol Pharm Bull.

[8] Peffault de Latour R, Fremeaux-Bacchi V, Porcher R (2015). Assessing complement blockade in patients with paroxysmal nocturnal hemoglobinuria receiving eculizumab. Blood.

[9] Dingli D, Matos JE, Lehrhaupt K, Krishnan S, Yeh M, Fishman J, Sarda SP, Baver SB (2022). The burden of illness in patients with paroxysmal nocturnal hemoglobinuria receiving treatment with the C5-inhibitors eculizumab or ravulizumab: results from a US patient survey. Ann Hematol.

[10] Cheng W, Sarda SP, Mody-Patel N, Krishnan S, Yenikomshian M, Scoble PJ, Mahendran M, Lejeune D, Yu L, Duh MS (2020). Real-world eculizumab dosing patterns among patients with paroxysmal nocturnal hemoglobinuria in a US population. Blood.

[11] Cheng W, Sarda SP, Mody-Patel N, Krishnan S, Yenikomshian M, Scoble PJ, Mahendran M, Lejeune D, Yu L, Duh MS (2020). Real-world treatment patterns and healthcare resource utilization (HRU) of patients (Pts) with paroxysmal nocturnal hemoglobinuria (PNH) receiving eculizumab in a US population. Blood.

[12] Kulasekararaj AG, Hill A, Rottinghaus ST (2019). Ravulizumab (ALXN1210) vs eculizumab in C5-inhibitor-experienced adult patients with PNH: the 302 study. Blood.

[13] Roth A, Rottinghaus ST, Hill A (2018). Ravulizumab (ALXN1210) in patients with paroxysmal nocturnal hemoglobinuria: results of 2 phase 1b/2 studies. Blood Adv.

[14] Dingli D, Matos JE, Lehrhaupt K, Krishnan S, Baver SB, Sarda SP (2020). Work Productivity loss and quality of life in paroxysmal nocturnal hemoglobinuria among patients receiving C5 inhibitors in the United States. Blood.

[15] Liao DS, Grossi FV, El Mehdi D (2020). Complement C3 inhibitor pegcetacoplan for geographic atrophy secondary to age-related macular degeneration: a randomized phase 2 trial. Ophthalmology.

[16] Hillmen P, Szer J, Weitz I (2021). Pegcetacoplan versus eculizumab in paroxysmal nocturnal hemoglobinuria. N Engl J Med.

[17] Peffault de Latour R, Szer, J, Weitz, I, Röth, A, Höchsmann, B, Panse, J, Usuki, K, Griffin, M, Kiladijan, J-J, de Castro, CM, Nishimori, H, Tan, L, Al-Adhami, M, Deschatelets, P, Francois, C, Grossi, F, Risitano, A, Hillmen, P. Forty-eight week efficacy and safety of pegcetacoplan in adult patients with paroxysmal nocturnal hemoglobinuria and suboptimal response to prior eculizumab treatment. Paper presented at: European Hematology Association 2021

[18] Cella DWK, Beaumont J (2003). *The FACIT-Fatigue Scale: Description, Reliability and Validity*.

[19] Weitz I, Meyers G, Lamy T (2013). Cross-sectional validation study of patient-reported outcomes in patients with paroxysmal nocturnal haemoglobinuria. Intern Med J.

[20] Aaronson NK, Ahmedzai S, Bergman B (1993). The European Organization for Research and Treatment of Cancer QLQ-C30: a quality-of-life instrument for use in international clinical trials in oncology. J Natl Cancer Inst.

[21] Hays RDRD, Fayers PHR (2005). Reliability and validity (including responsiveness). *Assessing Quality of Life in Clinical Trials: Methods and Practice*.

[22] Ebben JP, Gilbertson DT, Foley RN, Collins AJ (2006). Hemoglobin level variability: associations with comorbidity, intercurrent events, and hospitalizations. Clin J Am Soc Nephrol.

[23] Cohen J (1988). *Statistical Power for the Behavioral Sciences*.

[24] Keystone E, Burmester GR, Furie R (2008). Improvement in patient-reported outcomes in a rituximab trial in patients with severe rheumatoid arthritis refractory to anti-tumor necrosis factor therapy. Arthritis Rheum.

[25] Lai JS, Beaumont JL, Ogale S, Brunetta P, Cella D (2011). Validation of the functional assessment of chronic illness therapy-fatigue scale in patients with moderately to severely active systemic lupus erythematosus, participating in a clinical trial. J Rheumatol.

[26] Strand V, Burmester GR, Zerbini CA (2015). Tofacitinib with methotrexate in third-line treatment of patients with active rheumatoid arthritis: patient-reported outcomes from a phase III trial. Arthritis Care Res.

[27] Cella D, Lai JS, Chang CH, Peterman A, Slavin M (2002). Fatigue in cancer patients compared with fatigue in the general United States population. Cancer.

[28] Montan I, Lowe B, Cella D, Mehnert A, Hinz A (2018). General population norms for the Functional Assessment of Chronic Illness Therapy (FACIT)-Fatigue Scale. Value Health.

[29] Cella D, Eton DT, Lai JS, Peterman AH, Merkel DE (2002). Combining anchor and distribution-based methods to derive minimal clinically important differences on the Functional Assessment of Cancer Therapy (FACT) anemia and fatigue scales. J Pain Symptom Manag.

[30] Cella D, Yount S, Sorensen M, Chartash E, Sengupta N, Grober J (2005). Validation of the functional assessment of chronic illness therapy fatigue scale relative to other instrumentation in patients with rheumatoid arthritis. J Rheumatol.

[31] Brodsky RA (2014). Paroxysmal nocturnal hemoglobinuria. Blood.

[32] Vadhan-Raj S, Mirtsching B, Charu V (2003). Assessment of hematologic effects and fatigue in cancer patients with chemotherapy-induced anemia given darbepoetin alfa every two weeks. J Support Oncol.

[33] Alexander M, Kewalramani R, Agodoa I, Globe D (2007). Association of anemia correction with health related quality of life in patients not on dialysis. Curr Med Res Opin.

[34] Conlon NP, Bale EP, Herbison GP, McCarroll M (2008). Postoperative anemia and quality of life after primary hip arthroplasty in patients over 65 years old. Anesth Analg.

[35] Administration UFaD. Rare diseases: common issues in drug development, guidance for industry. 2019; https://www.fda.gov/regulatory-information/search-fda-guidance-documents/rare-diseases-common-issues-drug-development-guidance-industry-0. Accessed May 15, 2021

[36] Wang Y. Trial Design and Statistical Considerations in Rare Disease Clinical Trials. 2019; https://www.fda.gov/media/131882/download. Accessed May 15, 2021

[37] Niedeggen C, Singer S, Groth M (2019). Design and development of a disease-specific quality of life tool for patients with aplastic anaemia and/or paroxysmal nocturnal haemoglobinuria (QLQ-AA/PNH)-a report on phase III. Ann Hematol.

[38] Weisshaar K, Ewald H, Halter J (2020). Development of a patient-reported outcome questionnaire for aplastic anemia and paroxysmal nocturnal hemoglobinuria (PRO-AA/PNH). Orphanet J Rare Dis.

[39] Zini MLL, Banfi G (2021). A narrative literature review of bias in collecting patient reported outcomes measures (PROMs). Int J Environ Res Public Health.

