# Supplementary figures and images for: Changes in hemoglobin and clinical outcomes drive improvements in fatigue, quality of life, and physical function in patients with paroxysmal nocturnal hemoglobinuria: post hoc analyses from the phase III PEGASUS study
Source: Ann Hematol. 2022 Jul 23;101(9):1905–14. doi: 10.1007/s00277-022-04887-8 (PMC9375756; doi:10.1007/s00277-022-04887-8)

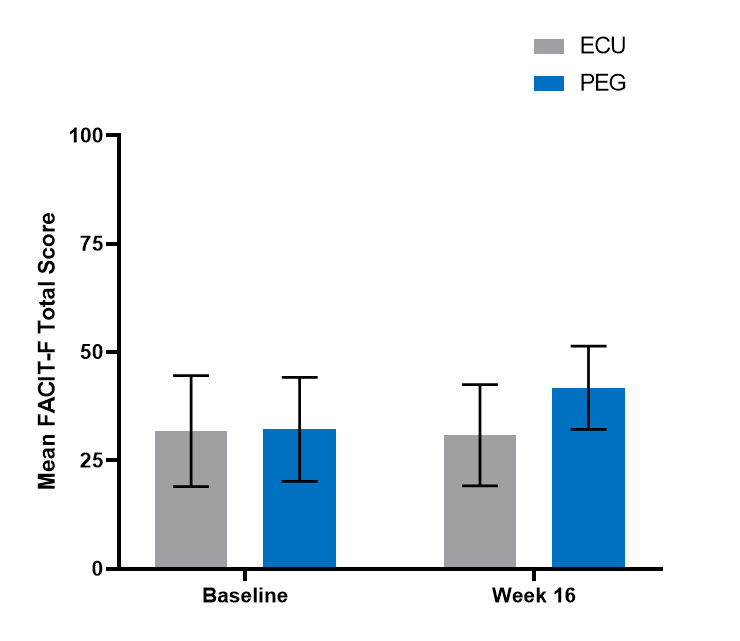

Supplement: Supplementary file 1 — (PNG 12 kb) [file 277_2022_4887_Fig6_ESM.png]

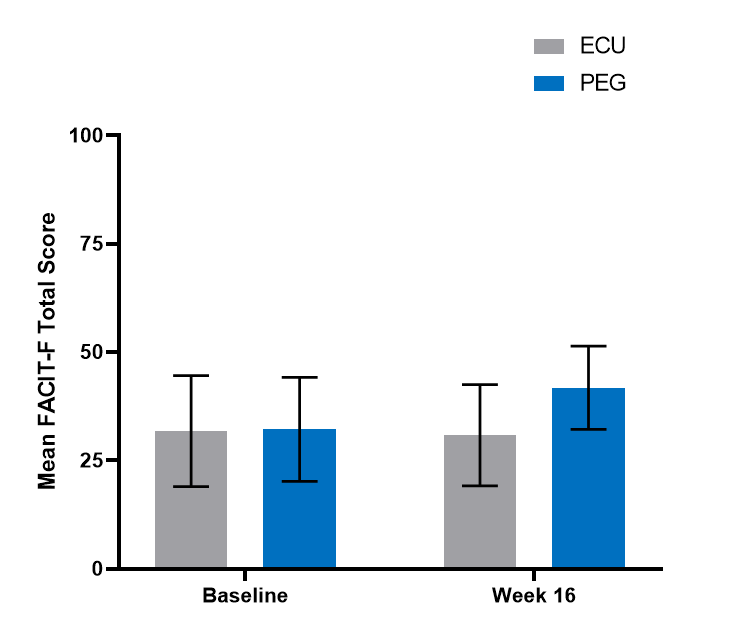

Supplement: Supplementary file 2 — High Resolution Image (TIF 24 kb) [file 277_2022_4887_MOESM1_ESM.tif]

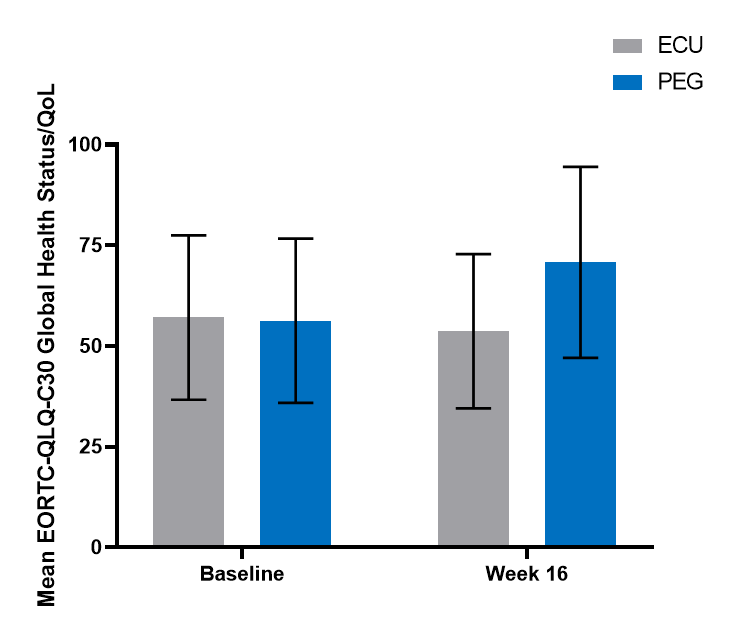

Supplement: Supplementary file 3 — (PNG 15 kb) [file 277_2022_4887_Fig7_ESM.png]

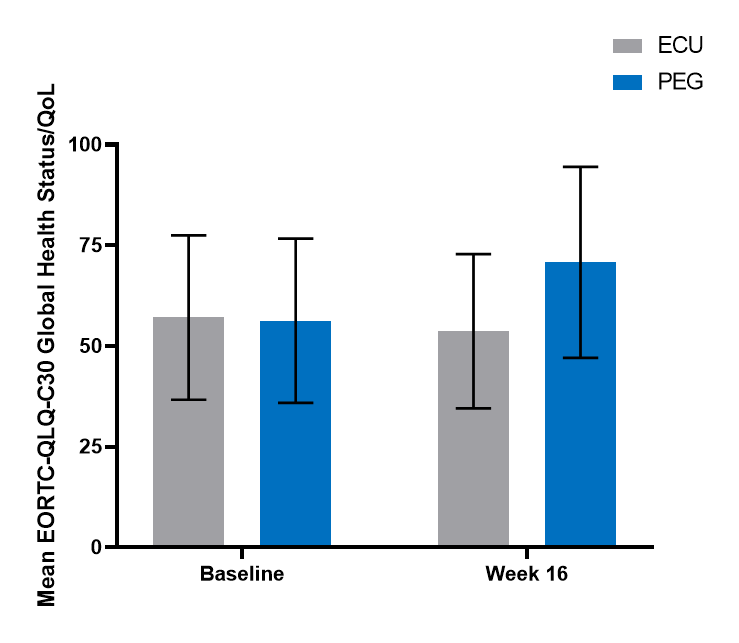

Supplement: Supplementary file 4 — High Resolution Image (TIF 29 kb) [file 277_2022_4887_MOESM2_ESM.tif]

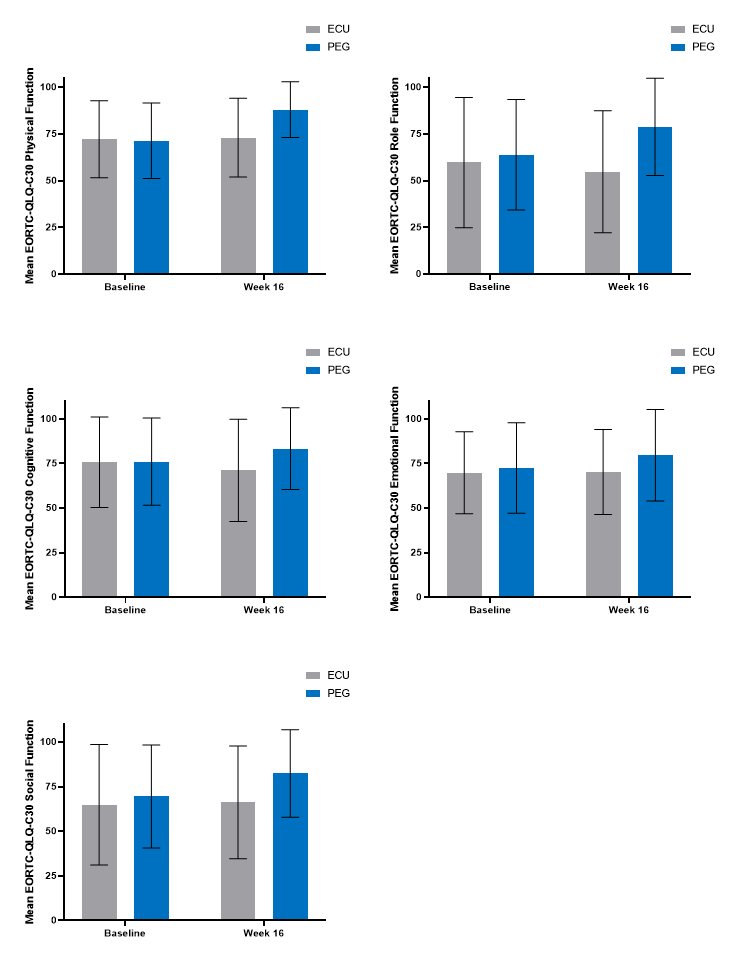

Supplement: Supplementary file 5 — (PNG 25 kb) [file 277_2022_4887_Fig8_ESM.png]

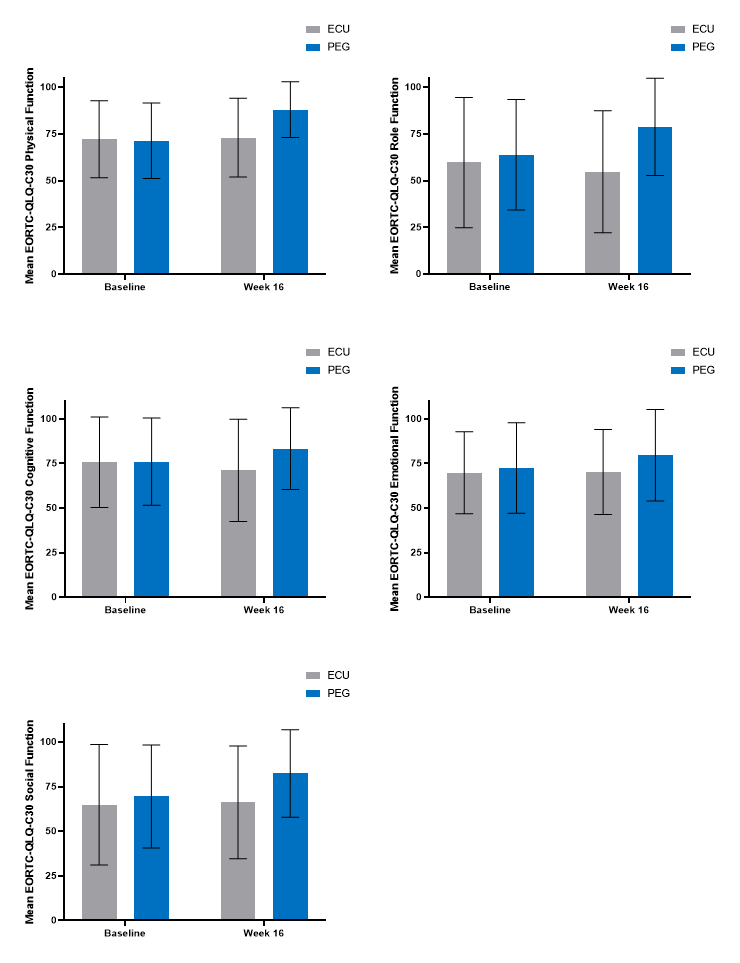

Supplement: Supplementary file 6 — High Resolution Image (TIF 59 kb) [file 277_2022_4887_MOESM3_ESM.tif]

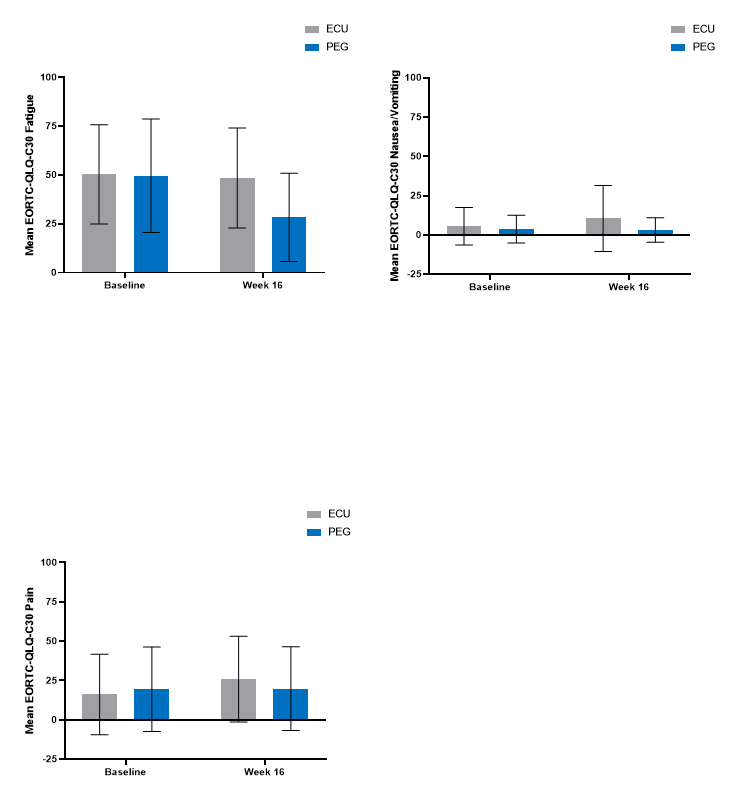

Supplement: Supplementary file 7 — (PNG 16 kb) [file 277_2022_4887_Fig9_ESM.png]

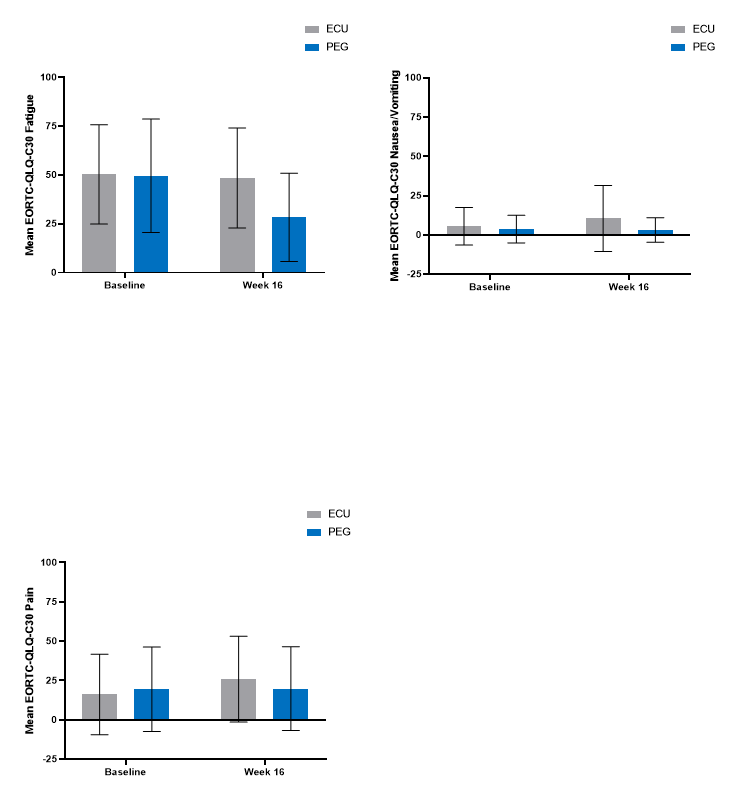

Supplement: Supplementary file 8 — High Resolution Image (TIF 32 kb) [file 277_2022_4887_MOESM4_ESM.tif]

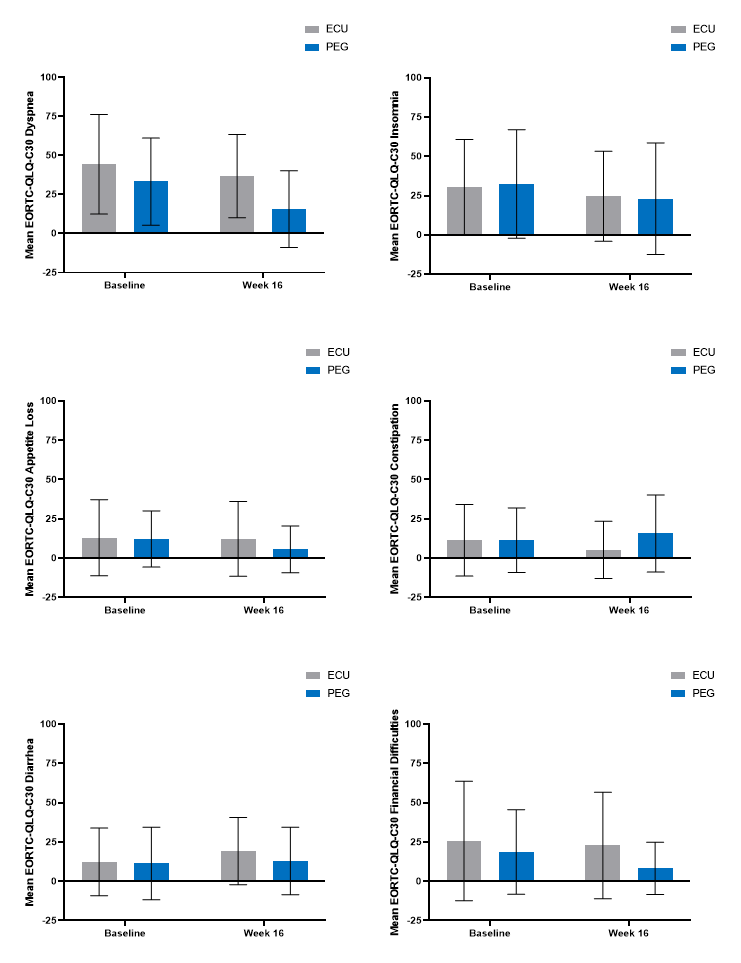

Supplement: Supplementary file 9 — (PNG 26 kb) [file 277_2022_4887_Fig10_ESM.png]

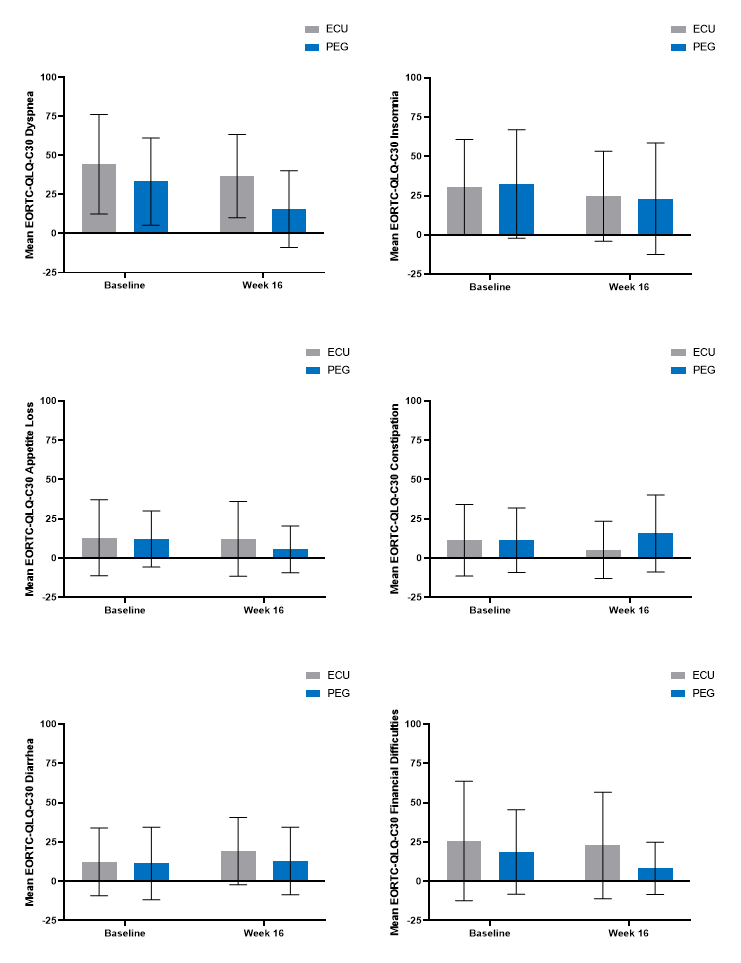

Supplement: Supplementary file 10 — High Resolution Image (TIF 55 kb) [file 277_2022_4887_MOESM5_ESM.tif]
